# Isolation and Metagenomic Identification of Avian Leukosis Virus Associated with Mortality in Broiler Chicken

**DOI:** 10.1155/2016/9058403

**Published:** 2016-08-15

**Authors:** Faruku Bande, Siti Suri Arshad, Abdul Rahman Omar

**Affiliations:** ^1^Department of Veterinary Pathology and Microbiology, Faculty of Veterinary Medicine, Universiti Putra Malaysia (UPM), 43400 Serdang, Selangor Darul Ehsan, Malaysia; ^2^Department of Veterinary Services, Ministry of Animal Health and Fisheries Development, PMB 2109, Usman Faruk Secretariat, Sokoto 840221, Sokoto State, Nigeria; ^3^Laboratory of Vaccine and Immunotherapeutic, Institute of Bioscience, Universiti Putra Malaysia (UPM), 43400 Serdang, Selangor Darul Ehsan, Malaysia

## Abstract

Avian leukosis virus (ALV) belongs to the family Retroviridae and causes considerable economic losses to the poultry industry. Following an outbreak associated with high mortality in a broiler flock in northern part of Malaysia, kidney tissues from affected chickens were submitted for virus isolation and identification in chicken embryonated egg and MDCK cells. Evidence of virus growth was indicated by haemorrhage and embryo mortality in egg culture. While viral growth in cell culture was evidenced by the development of cytopathic effects. The isolated virus was purified by sucrose gradient and identified using negative staining transmission electron microscopy. Further confirmation was achieved through next-generation sequencing and nucleotide sequence homology search. Analysis of the viral sequences using the NCBI BLAST tool revealed 99-100% sequence homology with exogenous ALV viral envelope protein. Phylogenetic analysis based on partial envelope sequences showed the Malaysian isolate clustered with Taiwanese and Japanese ALV strains, which were closer to ALV subgroup J, ALV subgroup E, and recombinant A/E isolates. Based on these findings, ALV was concluded to be associated with the present outbreak. It was recommended that further studies should be conducted on the molecular epidemiology and pathogenicity of the identified virus isolate.

## 1. Introduction

Avian leukosis virus (ALV) is an economically important retrovirus affecting meat and egg-type chicken. The virus belongs to an* Alpharetrovirus* genus in the family Retroviridae. Based on the envelope glycoprotein (gp85) it was possible to classify exogenous ALV into different subgroups, namely, A, B, C, D, E, and J. Particularly, the viral envelope glycoprotein is responsible for attachment and receptor specificity as well as the production of neutralizing antibodies [1, 2]. Of the viral subgroups so far identified, subgroups A, B, and J are considered most prevalent and more economically important [3]. Subgroup J was first isolated in meat-type chicken in the United Kingdom in 1989 but currently is causing devastation to poultry industry worldwide [4]. Apart from its immunosuppressive effect, ALV is commonly associated with lymphoid leukosis, myelocytic myeloid leukosis, and renal as well as other forms of tumours [5]. This study reports some virological and molecular sequencing approaches used to identify the viral cause of mortality in a broiler flock in Malaysia.

## 2. Materials and Methods

Following a suspected outbreak in a broiler chicken farm with capacity of about 10,000 birds, tissue samples, including trachea, kidney, and proventriculus, were submitted to the virology laboratory, Universiti Putra Malaysia, for virus isolation and identification. The mortality rate was reported to reach about 10% in the 27-day-old flock (*n* = 6000) and more than 20% in the 30-day-old flock (*n* = 4000). The chickens flock health programme consisted of vaccination against NDV, IBD, and IBV. Postmortem findings revealed mild petechial haemorrhage on the proventriculus and markedly swollen kidney (figures not shown). Samples were processed and inoculated in a 9-day-old embryonated chicken egg as well as MDCK cells and then monitored for the evidences of virus growth. Identification of the virus was carried out by electron microscopy using the negative staining methods while confirmation was done by next-generation sequencing using the MiSeq illumina sequencing platform. A ScriptSeq v2 RNA-Seq library preparation kit was employed and used according to manufacturer's guidelines (epicenter, USA).

## 3. Results and Discussion

**Figure 1:**
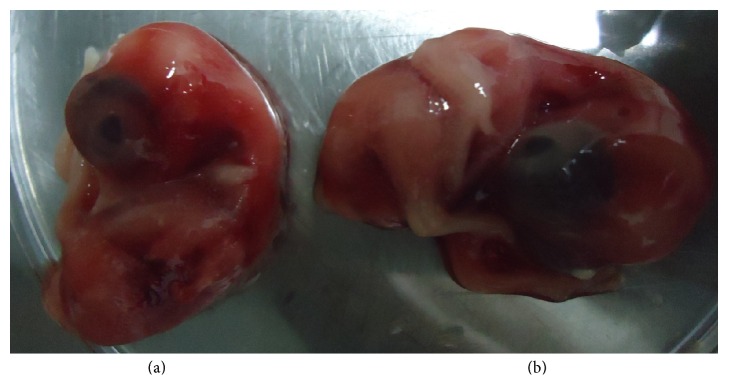
Inoculation of viral suspension in 9-day old embryonated chicken egg showing evidence of severe haemorrhage in infected embryo (a) as compared with negative control (b) after 24 hours pi.

Avian leukosis, specifically of subgroup J origins, has been reported previously in Malaysia [6]. This study investigated a viral cause of mortality observed in a broiler farm in northern Malaysia in 2013. Due to the involvement of kidney, initial tentative diagnosis focused on avian infectious bronchitis [7]; however inoculation of kidney suspension in chicken's embryonated egg revealed evidence of virus growth characterized by severe haemorrhage and embryo mortality (Figure 1). Embryo mortality was observed to increase as the passaging of virus increases to passage 3. Specifically, at passage 2, there was about 70%–100% mortality of embryos starting from day 2 to day 3 postinoculation (pi). Avian leukosis virus has been reported to cause severe haemorrhage and death of the embryo within 4 to 5 days following infection of egg embryo [5].

**Figure 2:**
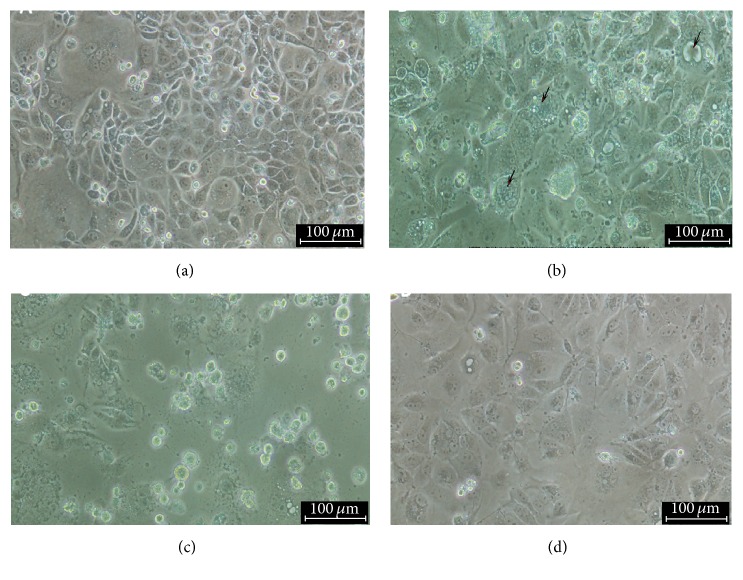
Infection of MDCK cells with virus suspension revealed evidences of virus growth as demonstrated by CPE which is characterized by cell ballooning, granulation, formation of giant cells (arrow), rounding, and cell detachment as infection progresses 3 dpi (a); 5 dpi (b) and 7 dpi (c); (d) control uninfected cells. Mag ×20 and 100 *μ*m.

Similarly, inoculation of MDCK cells with kidney derived suspension showed evidence of virus growth characterized by the presence of cytopathic effects including cell ballooning, granulation, rounding, giant cells formations, and cell detachment starting from day 3 pi (Figure 2). Although most ALVs produce no visible morphological changes in cell culture, infection of chicken embryo fibroblast cells with cytopathic ALVs strains such as the RAV-2 was reported to cause CPE and detachment of cells 3 days after infection [8]. Other studies confirmed the tropism and growth of ALVs in chicken embryo fibroblast cells based on the presence of CPE [9, 10]. It is also generally known that MDCK cells express receptors for some avian viruses such as avian influenza [11].

**Figure 3:**
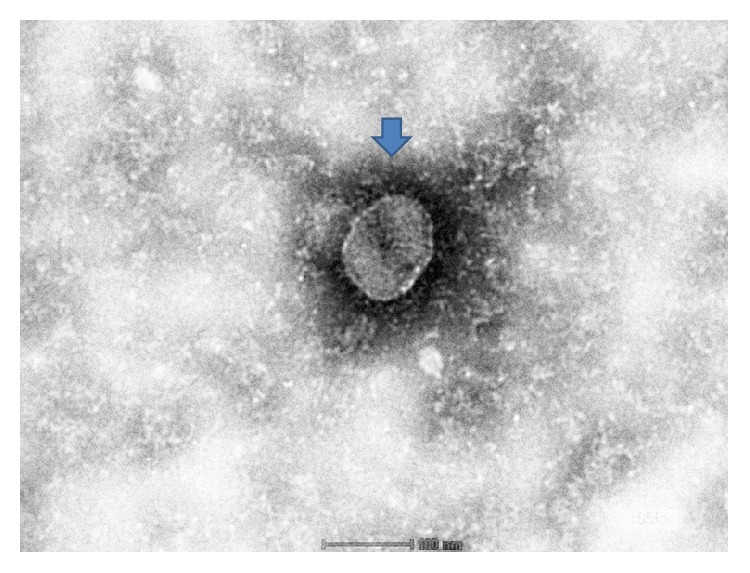
Negative staining electron microscopy showing the morphological appearance of the isolated virus. Note: presence of spike projections surrounding the viral particle (arrow) whose diameter ranges from 80 to 120 nm. Mag ×100 *μ*m.

Virus identification carried out using negative staining electron microscopy revealed a spherically rounded, virus-like particle with characteristic projections resembling spike structures (Figure 3). Subsequent analysis by comparison revealed that the virus morphology observed in this study was similar to that reported by Tsang et al. [12].

**Figure 4:**
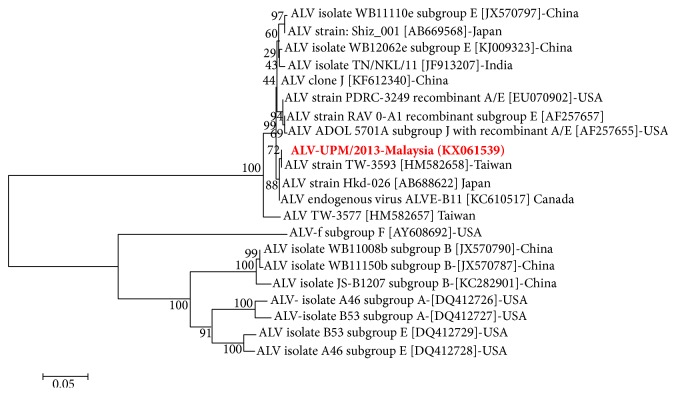
Neighbor-joining phylogenetic analysis using partial nucleotide sequences of the ALV envelope gp85 glycoprotein gene. Malaysian ALV isolate is presented in bold red ink. The confidence level of the inferred tree was determined using 1000 bootstrap. Evolutionary analysis was conducted in MEGA6 software.

Despite evidence of virus growth in egg embryo and cell culture, attempts to detect common viral diseases using specific primers against NDV, AI, and IBV were unsuccessful [7]; hence, a next-generation RNA sequencing approach was employed. Following sequencing, analysis of the generated sequences (Genbank accession number: KX061539) using NCBI BLAST revealed high sequence homology with exogenous ALV viral envelope protein region. Furthermore, phylogenetic characterization using the entire envelope sequences (1230 bp) revealed close identity with exogenous ALV-TW-3593 from Taiwan (100%), ALV-Hkd-026 strains from Japan (99%), and a putative endogeneous ALV sequence designated ALVE-B11 (99%). In terms of virus subgroup, the identified local ALV sequences demonstrated closeness to ALV J, ALV E, and recombinant ALV A/E as well as an endogenous ALV of chicken (Figure 4).

In terms of protein sequences, which comprise the receptor binding SU domain (gp85) and transmembrane protein TM domain (gp37), Malaysian ALV isolate also phylogenetically clustered with Taiwanese isolate (TW3593) and Chinese isolate (WB11008e) as well as Japanese isolate (Hkd026) (figure not shown).

In view of the nature of retroviruses, it is usual that mutation and recombination in ALV may influence virus diversity and tissue tropism as well as the severity of infection they cause [13]. These factors might probably account for the reported high mortality rate observed in the farm as well as during passaging of virus in embryo.

## 4. Conclusion

Based on the findings observed in this report, it was concluded that the present outbreak was associated with ALV. The study further demonstrates that ALV continues to circulate among poultry in Malaysia though it might be underdiagnosed. There is the need for flock monitoring against ALV and the possibility of including ALV infection as one of the differentials in related outbreaks. It is recommended to further study the molecular epidemiology and pathogenesis of the present local ALV strain in Malaysia.
